# Development of an Injectable Hydrogel for Histotripsy Ablation Toward Future Glioblastoma Therapy Applications

**DOI:** 10.1007/s10439-024-03601-1

**Published:** 2024-08-30

**Authors:** Zerin Mahzabin Khan, Junru Zhang, Jessica Gannon, Blake N. Johnson, Scott S. Verbridge, Eli Vlaisavljevich

**Affiliations:** 1 Virginia Tech – Wake Forest University School of Biomedical Engineering and Sciences, Blacksburg, VA 24061 USA; 2 Department of Biomedical Engineering, Yale University, New Haven, CT 06511 USA; 3 Department of Industrial and Systems Engineering, Virginia Tech, Blacksburg, VA 24061 USA; 4 Department of Biomedical Engineering and Mechanics, Virginia Tech, Blacksburg, VA 24061 USA

**Keywords:** Glioblastoma resection cavity, Thiol-Michael addition injectable hydrogel, Ultrasound imaging, Focused ultrasound, Histotripsy treatment, Red blood cell ablation

## Abstract

Glioblastoma (GBM) is the most common and malignant type of primary brain tumor. Even after surgery and chemoradiotherapy, residual GBM cells can infiltrate the healthy brain parenchyma to form secondary tumors. To mitigate GBM recurrence, we recently developed an injectable hydrogel that can be crosslinked in the resection cavity to attract, collect, and ablate residual GBM cells. We previously optimized a thiol-Michael addition hydrogel for physical, chemical, and biological compatibility with the GBM microenvironment and demonstrated CXCL12-mediated chemotaxis can attract and entrap GBM cells into this hydrogel. In this study, we synthesize hydrogels under conditions mimicking GBM resection cavities and assess feasibility of histotripsy to ablate hydrogel-encapsulated cells. The results showed the hydrogel synthesis was bio-orthogonal, not shear-thinning, and can be scaled up for injection into GBM resection mimics *in* *vitro*. Experiments also demonstrated ultrasound imaging can distinguish the synthetic hydrogel from healthy porcine brain tissue. Finally, a 500 kHz transducer applied focused ultrasound treatment to the synthetic hydrogels, with results demonstrating precise histotripsy bubble clouds could be sustained in order to uniformly ablate red blood cells encapsulated by the hydrogel for homogeneous, mechanical fractionation of the entrapped cells. Overall, this hydrogel is a promising platform for biomaterials-based GBM treatment.

## Introduction

Glioblastoma (GBM) is the most common and malignant type of primary brain cancer. Even after surgical resection and adjuvant chemoradiotherapy, residual tumor cells remaining in the resection cavity proliferate and migrate along white matter tracts to invade the healthy brain parenchyma [20]. Hence, secondary tumors form within 2–3 cm of the original border and lead to poor survival rates of less than 10% beyond 5 years [20]. To mitigate GBM recurrence, recent efforts have explored introducing biocompatible materials into the resection cavity for targeted anti-tumor effects. Biomaterials can spatiotemporally control release of therapeutic agents to bypass the blood-brain-barrier and reduce inhomogeneous delivery due to abnormal GBM vasculature [2]. Although these biomaterials, such as Gliadel wafers loaded with chemotherapeutics [43] deliver sufficient drug dosages, the material-tissue interface is hindered by their rigid shape preventing the entire cavity from being filled.

Compared to prefabricated materials, injectable hydrogels can be dispensed deeper into tissues [25] with minimum invasiveness according to patient-specific anatomical structures. Injectable hydrogels are three-dimensional (3D) polymeric, hydrophilic networks compatible with bodily fluids [22] for sol-gel transition *in situ*. Chemically crosslinked hydrogels are synthesized through new covalent bonds between polymer chains, and compared to physical crosslinking, they have more stable crosslinked points, slower degradation rates, and longer stability for increased mechanical strength [27, 44]. Injectable hydrogels in cancer therapy have been extensively studied [13], as direct injection ensures physical proximity to tumors for effective, local anti-tumor effects with enhanced material-tissue interface and engagement with cells [1].

Recent research has exploited GBM cells’ invasive potential by redirecting their migration for subsequent eradication via cytotoxic therapies. For example, Autier and colleagues developed a nanofibrous, bacterial cellulose hydrogel to release chemoattractants to attract and adhere F98 glioma cells on the hydrogel surface [3]. Safi and colleagues developed an alginate - chitosan hydrogel to passively capture F98 cells within macroporous hydrogel matrices [31]. While GBM cells can be entrapped into these biomaterials, the hydrogels were not injectable or degradable. Degradable, injectable materials are advantageous for GBM therapy, since they minimally compress the brain parenchyma and bypass invasive surgical procedures for removal post-treatment [1]. Furthermore, both research efforts propose stereotactic radiosurgery for ablating hydrogel-entrapped cells. GBM ablation via stereotactic radiosurgery has not significantly improved patient survival [30] due to limitations, such as radioresistant phenotypes of GBM cells possessing DNA damage repair mechanisms that can evade radiation therapy, which can further enrich therapy-resistant subtypes [21].

To address these limitations, we are developing an injectable, biodegradable hydrogel to attract and entrap GBM cells with chemoattraction for their subsequent ablation. We envision after surgical resection, a hydrogel preloaded with chemokines can be injected into the resection cavity. Chemokine release can induce GBM invasion into the hydrogel, and focused ultrasound (FUS) can then non-invasively and mechanically ablate entrapped cells. We had previously characterized a thiol-Michael addition hydrogel and demonstrated the formulation comprising 0.175 M NaHCO_3(aq)_ and 50 wt% water content was the most physically, chemically, and biologically compatible with the GBM microenvironment and our proposed treatment [19]. This hydrogel is customizable, forms well crosslinked networks, and is biocompatible with healthy brain cells like astrocytes [19].

We had previously demonstrated *in vitro* that CXCL12 chemokines released from this hydrogel induced GBM cell invasion from the extracellular matrix (ECM) and into the hydrogel via ameboid migration [17]. In this study, we develop our hydrogel further by scaling up the synthesis *in vitro* for injection into GBM resection mimics and assess the feasibility of ablating cells entrapped in the hydrogel. We previously characterized the storage and loss moduli of the swelled synthetic hydrogel with a rheometer [19]. However, the impact of injection force during synthesis, which can be regulated by controlling flowrates, is also important to consider [6]. The injection force and flowrate are impacted by the needle gauge during extrusion, and a reduction in the needle diameter increases the flowrate and thereby the injection force when an injectable hydrogel is dispensed through a syringe [16]. The viscosity of shear-thinning hydrogels decreases due to shear force during extrusion and increases once the shear stress is removed for sol–gel transition after injection [6]. In our current work, we utilize cantilever sensors to obtain critical viscoelastic material properties of the crosslinked hydrogels directly after injection through three different nozzle sizes and flowrates. These measurements were performed to assess hydrogel responses to alterations in shear stress and thereby viscoelastic properties during injection without requiring the post-processing steps necessary with rheometry [14].

Ultrasound imaging can be used for GBM diagnostic and therapeutic applications like monitoring cerebral blood flow, ablation, and tumor sizes [29]. While thiol-acrylate hydrogels can be distinguished from water and tissue with magnetic resonance imaging and computerized tomography imaging [28], these modalities are expensive and require patient transportation. Ultrasound imaging is relatively inexpensive and can be administered bedside with FUS for real-time guidance during targeted ablation [29]. Hence, we assess the hydrogel response to ultrasound imaging and histotripsy, which is an emerging, non-invasive FUS therapy that is promising for brain tumor treatment [45]. Since human skulls attenuate ultrasound transmission by reflection or absorption [12], we also assess ultrasound imaging through a polyolefin-based cranial implant [29] which can reduce attenuation of ultrasound waves to improve imaging through the skull. As histotripsy ablation is non-thermal and non-ionizing, it can bypass many limitations encountered by radiotherapy and thermal ablation. In this study, intrinsic threshold histotripsy was applied to treat the hydrogels, since compared to contrast agents or long FUS pulses, it results in more reproducible and predictable cavitation clouds with high ablation efficiencies at high peak negative pressures (*p*−) [10, 23]. Cerebrospinal fluid will diffuse into implanted hydrogels, and we had previously demonstrated that the synthetic hydrogel swelling impacts many of its properties, including its viscoelastic properties and degradation kinetics [19]. Hence, we evaluate the impact of hydrogel swelling on the cavitation bubble cloud characteristics during histotripsy treatment and assess the feasibility of histotripsy to mechanically ablate hydrogel-entrapped cells toward our proposed GBM therapy.

## Materials and Methods

### Dual Chamber Syringe Mixer with Adjustable Nozzle

**Fig. 1:**
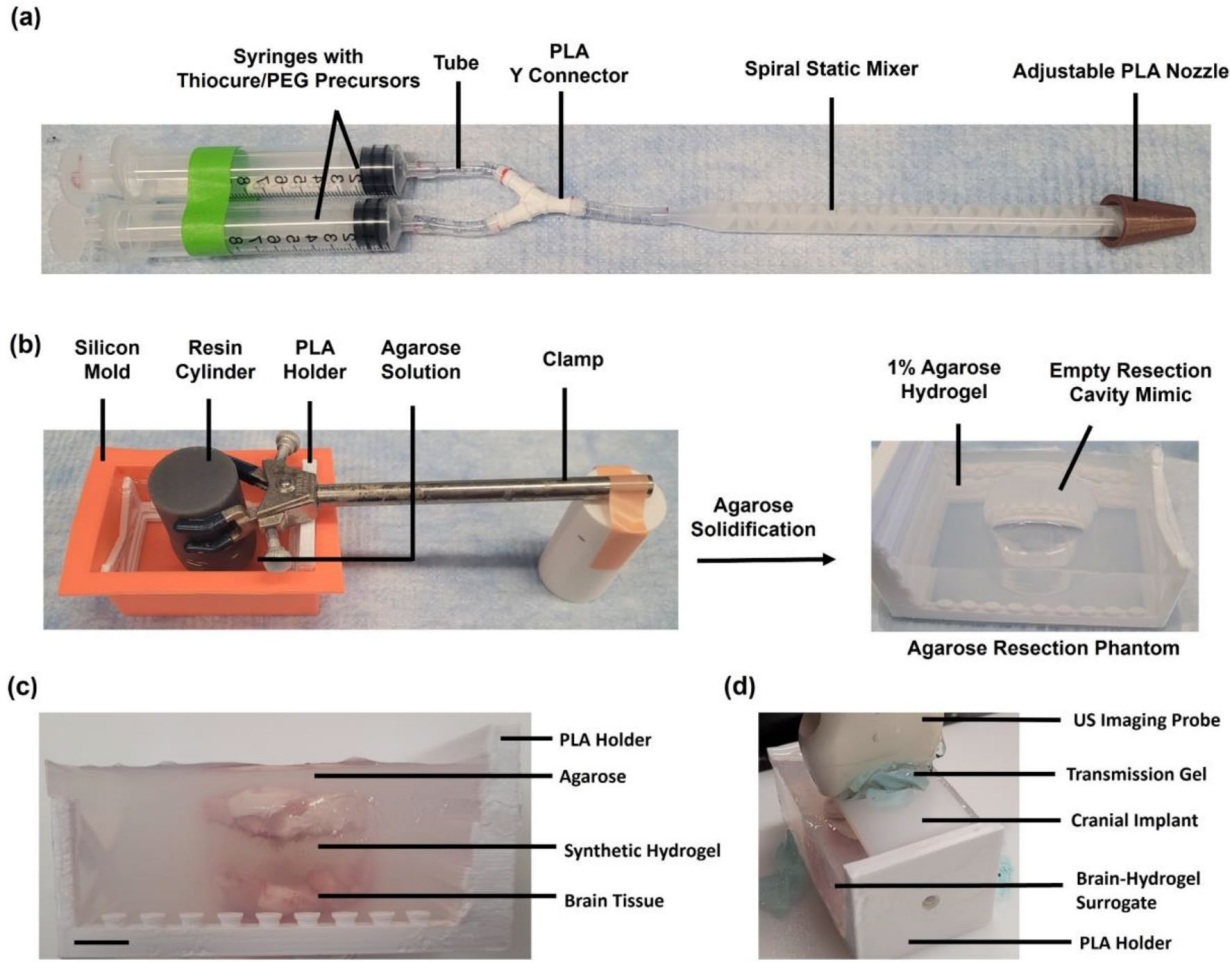
Setup for thiol-Michael addition hydrogel synthesis at clinically relevant volumes. **A** Dual chamber syringe mixer for synthesizing hydrogels upon injection. Precursor solutions were separately preloaded into each syringe barrel at equal volumes. The plungers were pushed simultaneously to mix the solutions in a 1:1 ratio through the Y connector tube and static mixer before dispensing through the adjustable nozzle. **B** Development of agarose resection phantoms. PLA holder frames were placed in a silicon mold. A resin printed solid cylinder was positioned over the PLA frame with a clamp while 1% agarose solution was poured into the PLA holder. After gelation, the cylinder was removed to generate a cavity in the agarose hydrogel that measured 3 cm in width and 2.55 cm in height. **C** Healthy porcine brain tissue surrogates for ultrasound imaging without cranial implant. The probe was positioned on top of the porcine brain tissue surrogates in agarose resection phantoms. Scale bar is 1 cm. **D** Ultrasound imaging of porcine brain tissue surrogates through cranial implant. The cranial implant was placed over the top of the porcine brain tissue surrogates and the ultrasound imaging probe was positioned over the cranial implant to obtain B-scan images of the surrogate through the implant.

Synthetic hydrogels were synthesized with a dual chamber syringe mixer (Figure 1a), which was adapted from a previous design [28] to adjust the nozzle diameter and flowrate during injection. Polyvinyl chloride tubes (Fisher Scientific) with 1/8 inch inner diameter (ID) were attached to two luer slip syringes and a polylactic acid (PLA) 3D printed Y-connector. Tubing connected the Y-connector to one of two spiral static mixers. The static mixer (ConProTec) for synthesizing hydrogels greater than 5 mL had length of 130 mm with 1/4 inch ID. The static mixer (Good News) for synthesizing hydrogels less than 5 mL had 0.38 inch ID with 58 mm length. Static mixers were connected to PLA 3D printed cone shaped nozzles with ID of 2.1 (small), 4.1 (medium), or 6.1 (large) mm.

### Synthetic Hydrogel Synthesis

Thiol-Michael addition hydrogel synthesis was adapted from our previous protocol [17, 19]. Thiocure 333L (ethoxylated trimethylolpropane tri-3-mercaptopropionate trithiol) was donated by Bruno Bock Thiochemicals. Thiocure and 575 g/mol poly(ethylene glycol) diacrylate (PEGDA) (Sigma-Aldrich) were brought to room temperature. NaHCO_3(aq)_ (Fisher Chemical) at 0.175 M was prepared in deionized water. For 10 mL hydrogels in a 1:1 acrylate:thiol stoichiometric ratio, 3.890 g of Thiocure, 3.000 g of PEGDA, and 6.9 mL of 0.175 M NaHCO_3(aq)_ were combined. Ratios were scaled appropriately for different final hydrogel volumes. NaHCO_3(aq)_ solution was split to dissolve the precursors (PEGDA and Thiocure) equally and vortexed for 30 s, then degassed in a vacuum desiccator for 30 min at room temperature and 20 psig to remove any bubbles. Each solution was separately loaded into each barrel in the syringe device, and plungers were pushed simultaneously to mix the precursors, then the solution was injected through the nozzle.

### Agarose Synthesis and Agarose Resection Cavity Phantoms

While healthy human brain tissues are approximately 1 kPa, GBM is associated with high intracranial pressures and compression stiffening of the tissues due to increased GBM vascularization and ECM remodeling as a result of higher secretion of ECM components by glioma cells in comparison to healthy brain cells [4, 7]. Some GBM tissue stiffnesses have been reported to be as high as around 25 kPa [41]. As such, agarose hydrogels were prepared at 1% (w/v) concentrations, which possess a 21.7 kPa stiffness [39] and may mimic the human brain as reported by neurosurgeons [32]. While the actual stiffness of human brain may differ from this value, the 1% agarose phantoms were chosen as a model resection cavity for our initial study. Future work will be needed to more fully investigate resection cavities with varied stiffness as well as more clinically relevant characteristics that may include more optimal vascularization and tissue mimicking characteristics (i.e. cellularized scaffolds with more relevant anatomical features).

Agarose synthesis was adapted from our previous protocol [10]. Low melting point UltraPure agarose powder (Invitrogen) was dissolved in degassed 0.9% NaCl_(aq)_, heated by flash boiling, and degassed for 30 min in a vacuum desiccator at 20 psig and room temperature to cool to 40 °C and yield 1% (w/v) agarose. Agarose resection phantoms were developed to mimic the *in vivo* dimensions of GBM resection cavities and recapitulate *in situ* crosslinking of the synthetic hydrogels under clinically relevant volumes. A 3D printed resin (FormLabs Grey Pro) solid cylinder (5 cm height and 3 cm diameter) was positioned with a clamp over a PLA 3D printed (Crealty Ender 3 Pro) holder placed inside a rectangle silicon mold (Fig. 1b) to form an agarose layer at the bottom of the cavity. 75 mL of 1% degassed agarose solution was pipetted into this mold and crosslinked at 4 °C for 1 hour. The cylinder was removed to generate the cavity in the agarose which measured 2.55 cm in height and 3 cm in diameter (Fig. 1b), since primary GBM tumors are typically 3 cm wide [33]. Median GBM resection cavity volumes *in vivo* range from 21.7 to 26.6 mL [11]. To account for 30% hydrogel swelling due to fluid diffusion as we had determined previously [19], the resection phantoms were designed to hold clinically relevant hydrogel volumes of 18 mL.

### Impact of Swelling and Nozzle Size on Hydrogel Viscoelastic Properties

Hydrogels were prepared at 4 mL volumes. For swelled hydrogels, three sets were separately synthesized with the syringe mixer using medium nozzles, injected into three wells in a 48 well plate, and crosslinked at 37 °C for 30 min. Well plates were submerged in 400 mL of 1X phosphate buffered saline solution (PBS) at 37 °C for 24 hours. Cured hydrogels were similarly prepared in triplicate for each nozzle size without swelling in PBS.

Piezoelectric-excited millimeter cantilever (PEMC) sensors quantified the real-time responses of the hydrogel viscoelastic properties through fluid-structure interaction effects [35]. We recently showed the phase angle response of resonant piezoelectric milli-cantilevers correlates with the viscoelastic properties of several hydrogels [5, 14, 46]. At resonance, the phase angle indicates the relative displacement of the piezoelectric layer and is hence an indirect measure of the sensor’s amplitude [14]. The resulting sensor data in terms of phase angle is inversely proportional to the storage modulus [14], and samples with higher phase angle and phase angle shifts are less stiff than samples with lower phase angle and phase angle shifts. Since these cantilever sensors can detect persistent resonance in hydrogels, including PEGDA-based hydrogels, they can quantify the viscoelastic properties even after the sol–gel transition of hydrogels [14].

The sensors were fabricated as described previously [14]. The sensor phase angle at resonance was monitored over time through electromechanical coupling using a vector network analyzer with electrical impedance analysis (E5061b-005 Keysight) and a custom MATLAB program. Sensor impedance spectra were collected in air for 3 min to stabilize the sensor, and then the probe was positioned 4 mm deep into the middle of the hydrogel to obtain data over 7 min. The corresponding phase angle shifts were determined by subtracting the average phase angle in air from average phase angle in the hydrogel.

### Hydrogel Bio-orthogonality

The synthetic hydrogel’s bio-orthogonality and ability to crosslink in fluids during injection was assessed, as 14 mL of cerebrospinal fluid typically fills up the GBM resection cavities [33]. 14 mL of 1X PBS containing Ca^2+^/Mg^2+^ ions (Fisher Scientific) at 37 °C was added into a 6-dram vial (Fisher Scientific). In triplicate, precursors were prepared for 4 mL hydrogel volumes. 10 µL of blue food color dye (Kroger) was mixed into the PEGDA precursor solution by vortexing for 30 s. After degassing, precursor solutions were loaded into the syringe mixer with the medium nozzle. The hydrogel was injected into the PBS, incubated at 37 °C for 5 min, removed, and then placed on a watch glass to assess its uniformity.

### Ultrasound Imaging of Synthetic Hydrogel

Three agarose resection cavity phantoms were prepared. Whole healthy porcine brains obtained from a local slaughterhouse (Gunoe Sausage) were kept frozen until use, at which point they were thawed to room temperature and brain tissues were sliced off. Hydrogel-brain tissue surrogates were prepared by encasing a layer of synthetic hydrogel between two layers of porcine brain tissues in the resection cavity. 3.8 g of porcine brain tissues were placed on the bottom of the agarose resection cavity, and 5 mL of the synthetic hydrogel was injected on top of the brain tissues and crosslinked for 20 min at 37 °C. 5.2 g of porcine brain tissues were placed on top of the crosslinked synthetic hydrogel. 20 mL of degassed 1% agarose solution was placed on top of the brain tissue and solidified for 30 min at 4 °C. Each layer of the brain tissue surrogate is outlined in Fig. 1c. The ultrasound imaging probe (Telemed L18-10L30H-4) was positioned over a layer of transmission gel (Aquasonic) on top of the surrogate surface prior to imaging. The focus was set to 20–39 mm, depth to 40 mm, dynamic range to 72 dB, gain to 100%, and frequency to 14 MHz for optimal resolution and depth. For each surrogate, the ultrasound imaging was repeated through a polyolefin-based cranial window prosthesis (3 × 6 cm with 4 mm thickness) developed by Prada and colleagues [29] to assess the feasibility of low-cost chronic ultrasound imaging (Fig. 1d). B-scan images were obtained using the probe with Echo Wave II and OBS studio software. The imaging probe was oriented in the same plane and position for B-scan images with and without the cranial implant to directly compare these images.

### Histotripsy Cavitation Threshold

Three replicates of agarose resection cavity phantoms were prepared, and synthetic hydrogels were injected into each cavity with the syringe mixer at 18 mL volumes and crosslinked for 30 min at 37 °C. An agarose only control was also prepared without a cavity. The synthetic hydrogels (non-swelled) and agarose hydrogels (cured) were subjected to histotripsy cavitation threshold studies immediately. For swelled synthetic hydrogels, three replicates were prepared as described. A control agarose only without a cavity was also prepared (hydrated agarose), and all of these hydrogels were submerged in 300 mL of 1X PBS at 37 °C for 24 hours prior to histotripsy treatment.

**Fig. 2:**
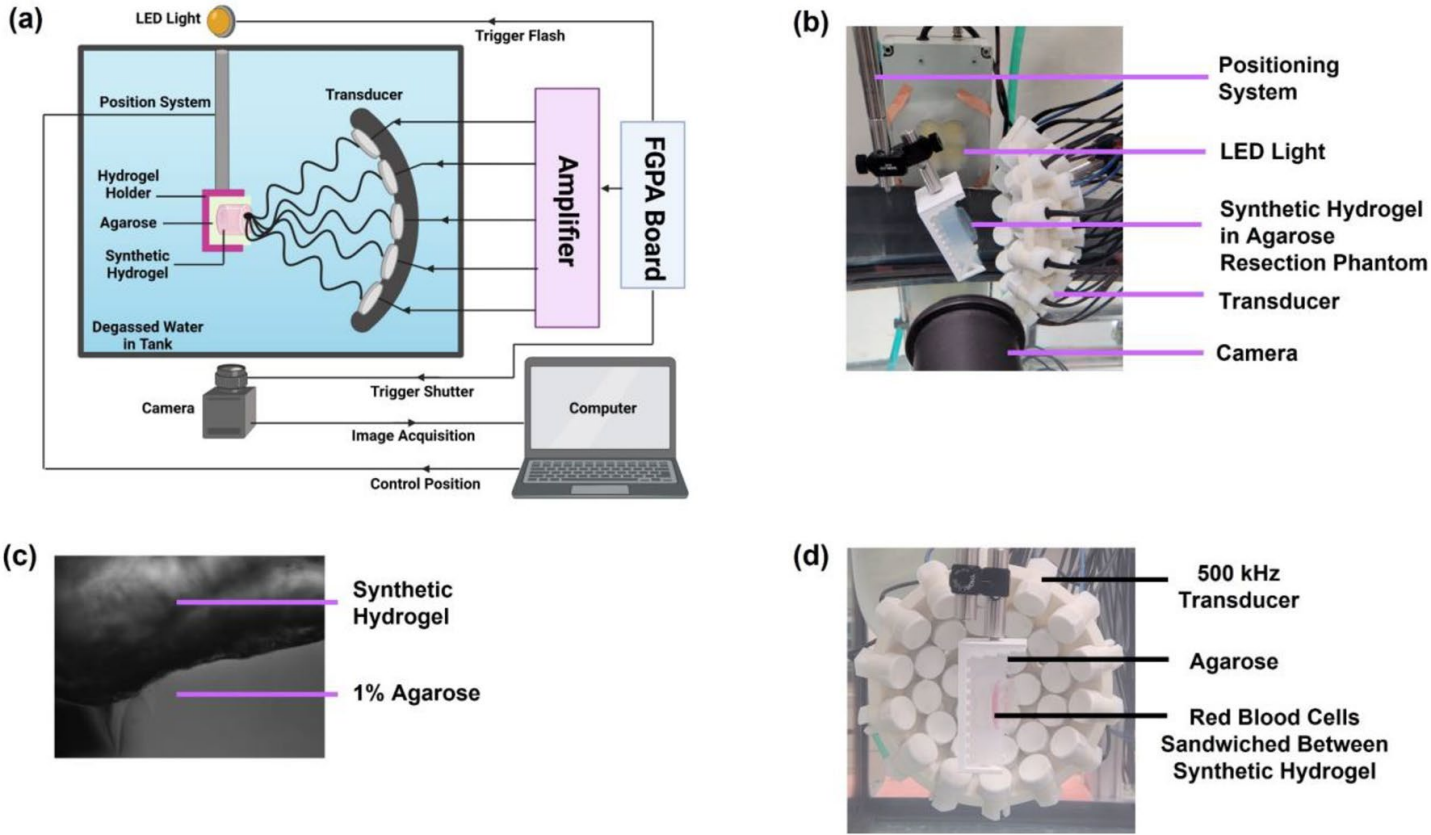
Histotripsy setup for focused ultrasound treatment of synthetic hydrogels in agarose-hydrogel resection phantoms. **A** Schematic overview of histotripsy setup, including the field-programmable gate array (FGPA) board connected to the amplifier that supplied the histotripsy pulsing to the transducer through high voltages. **B** Synthetic hydrogel in agarose resection phantoms subjected to histotripsy cavitation with 500 kHz transducer. **C** Synthetic hydrogels were distinguishable from 1% agarose in the resection cavity by a difference in opacity and uniformity in hydrogel layers. **D** Red blood cell ablation setup. Red blood cells were suspended in agarose in 1 mm thick layer that was sandwiched between two synthetic hydrogel layers within the agarose resection cavity mimics. The RBC layer was positioned at the focal point of the transducer and placed parallel to the transducer axis to enable the camera to visualize RBC ablation during treatment.

The FUS setup (Fig. 2a) was adapted from our previous protocol [10]. A water tank with filtered and degassed water was used to submerge an in-house built 500 kHz array transducer comprising 32 elements with a 120.5 mm aperture, 75 mm focus, and 0.62 f-number [10]. The agarose-synthetic hydrogels were attached to a three-axis motorized positioning system controlled by MATLAB and positioned at the transducer focus. A high-speed camera (FLIR Blackfly) with a 100 mm F2.8 Macro lens (Tokina) was positioned orthogonally to the transducer and parallel to the hydrogel to capture images at a resolution of 3.25 µm per pixel during histotripsy treatment (Fig. 2b). A custom designed LED strobe light backlit hydrogels to visualize single bubbles as dark shadows and was set to trigger at a delay of 8 µs after each pulse to capture the fully expanded bubble cloud [10]. An intensity threshold based on the background intensity, as optimized previously [23], was utilized to convert the acquired images from gray scale to binary, with bubbles detected as black regions greater than 5 pixels. The synthetic hydrogel with striations was distinguishable from the agarose layer (Fig. 2c). The transducer output pressure pulses were controlled by MATLAB and driven by a custom-built amplifier powered through a high voltage source to produce single cycle pulses.

A pulse repetition frequency (PRF) of 0.5 Hz was used to minimize bubbles generated with one cavitation pulse from affecting the probability of cavitation occurring in the subsequent pulse [39]. 100 histotripsy pulses were applied at each *p*- varied incrementally from 5.4 to 49.9 MPa to identify the cavitation threshold for each sample. The focal position within the synthetic hydrogel was changed with each *p*− to avoid treating the same region more than once. Calibration of the transducer focal pressures was conducted with a fiber optic hydrophone as described previously [10] to convert the voltages to *p*−. The acquired images were converted to binary from gray scale based on an intensity threshold determined by the background using a custom MATLAB code [10]. MATLAB was used to quantify bubble sizes and bubble cloud sizes (above the cavitation threshold) and the cavitation threshold. Bubbles were identified as black regions larger than 5 pixels in diameter, while the cavitation probability was defined as the fraction of pulses out of 100 pulses that yielded a bubble at a specific *p*−. The technique previously developed [39] and employed [10] was used to curve fit the cavitation probability to a sigmoidal function based on the error function to identify the cavitation thresholds, which were defined as *p*− at a single histotripsy pulse that corresponded to a cavitation probability of 0.5. A custom MATLAB code determined pulse-to-pulse coefficient correlation of bubbles to quantify persistent bubbles located in same region between pulses. Bubble cloud characteristics were quantified by measuring the bubble diameters, bubble cloud sizes, and cavitation thresholds for non-swelled and swelled hydrogels.

### Histotripsy Ablation of Red Blood Cells

Agarose resection phantoms containing a RBC layer entrapped between two layers of synthetic hydrogels were developed and subjected to histotripsy in order to evaluate the feasibility of ablating cells entrapped by the synthetic hydrogel with treatment. The RBC ablation can be monitored in real-time by imaging, since the cells undergo a change in opacity from opaque to transparent upon lysis [24]. Agarose resection phantoms were prepared, synthetic hydrogels at 13 mL were injected into the cavity with the syringe mixer, and hydrogels were crosslinked at 37 °C for 10 min. The anticoagulant citrate-phosphate-dextrose solution (Sigma-Aldrich) was mixed with porcine whole blood (Virginia Tech Meat Center) in a ratio of 9:1 (v/v) of blood to anticoagulant, stored at 4 °C, and used within 14 days. RBCs were isolated from whole blood by centrifugation and suspended in 1% agarose solution as described previously [10] at 5% (v/v) of RBC and stirred gently to mix. This RBC-agarose mixture was dispensed into the resection cavity on top of the synthetic hydrogel layer to form a 1 mm thick RBC layer and solidified for 10 minutes at 4 °C. Another layer of synthetic hydrogel was injected on top of the RBC layer to fill the cavity and crosslinked at 37 °C for 10 min. Samples were prepared in triplicate and subjected to histotripsy immediately. Each synthetic hydrogel layer was at least 5 mm thick to ensure the full axial length of the bubble cloud encompassed only the synthetic hydrogel and RBC layers.

The RBC layer was positioned parallel to the transducer axis at the focal point (Fig. 2d) to track RBC lesion area using the camera, which captured two images for each pulse, including an image of the bubble cloud and another image after the pulse for the lesion area without bubbles. Histotripsy was applied for 500 pulses with 0.5 Hz PRF at 48 MPa *p*− for an exposure of 16 min and 40 s. For each sample, three different regions of the RBC layer were treated, and lesion areas were quantified with the ImageJ polygon tool and normalized to the total ablation area at the 500th pulse to determine the percentage of area ablated per pulse.

## Results

### Hydrogel Synthesis and Bio-orthogonality

**Fig. 3:**
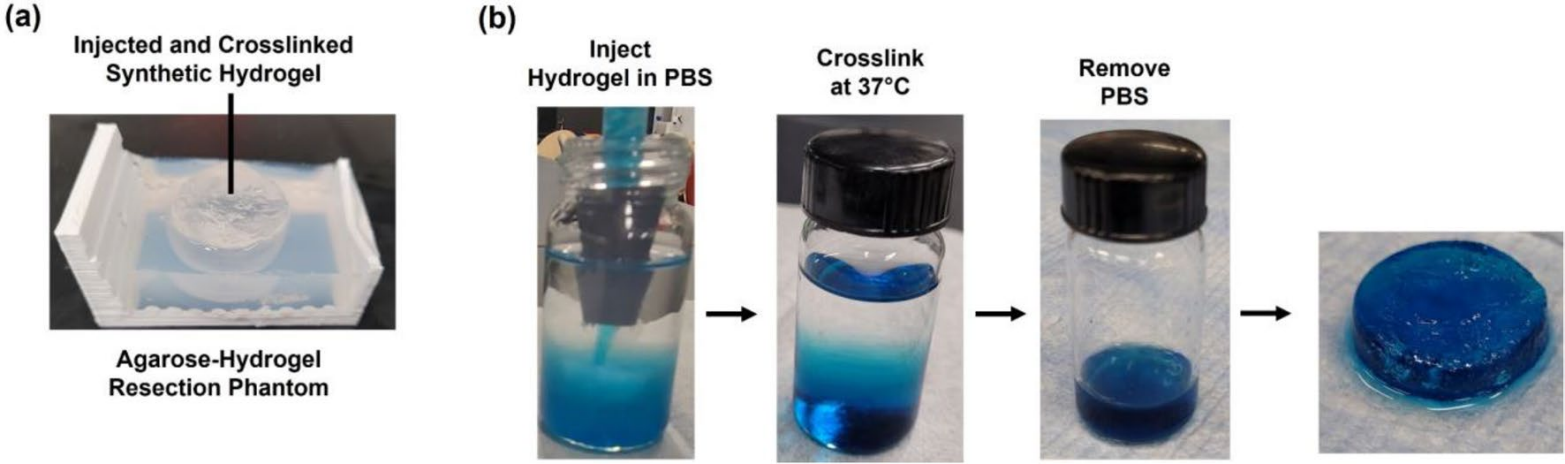
Thiol-Michael addition hydrogel synthesis. **A** The synthetic hydrogel was injected and crosslinked directly into the agarose resection cavity by using the dual chamber syringe mixer. **B** Synthetic hydrogel bio-orthogonality. In triplicate, synthetic hydrogels were injected into 1X PBS. PEGDA precursor solutions were mixed with blue food color dye to visualize the hydrogel during crosslinking. Hydrogels were crosslinked at 37 °C and sol–gel transition was assessed qualitatively.

The thiol-Michael addition hydrogel synthesis by injection through the dual chamber syringe mixer led to uniformly crosslinked hydrogels in the resection phantoms (Fig. 3a) without introducing bubbles, without clogging the nozzle, and crosslinking within 41 s in a clinically relevant timeframe [19]. The hydrogel synthesis was bio-orthogonal, as direct injection into PBS resulted in minimal to no dispersion with uniformly crosslinked hydrogels that were solid and not amorphous (Fig. 3b).

### Impact of Nozzle Size and Swelling

**Fig. 4:**
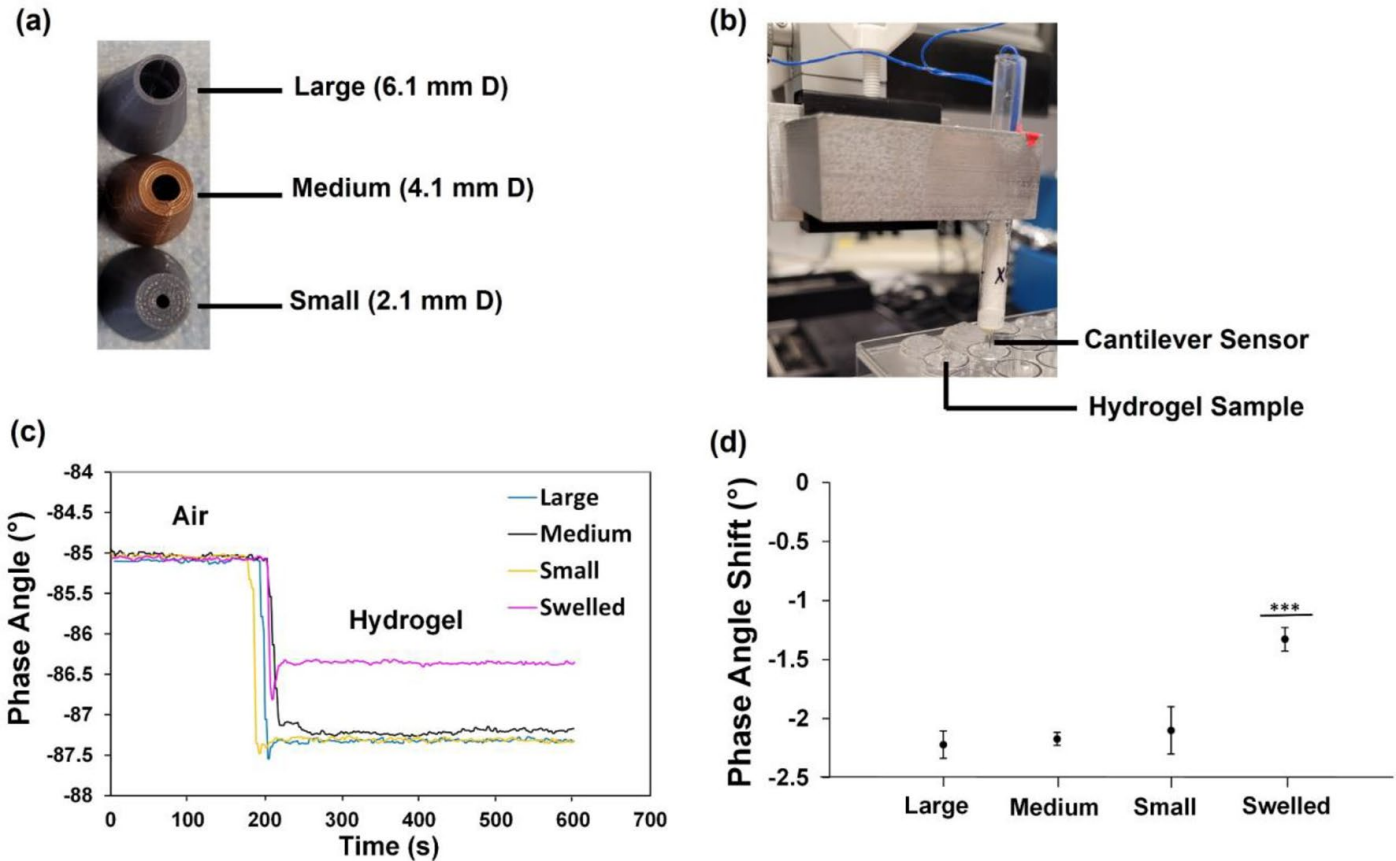
Impact of dual chamber syringe mixer nozzle size and hydrogel swelling on synthetic hydrogel viscoelastic properties as determined by cantilever sensors. **A** PLA 3D printed nozzles of various inner diameters for injecting synthetic hydrogels. **B** Piezoelectric-excited millimeter cantilever sensors were submerged into synthetic hydrogels. **C** Phase angle responses of the sensors first in air for 3 min and then submerged in the hydrogels for 7 min. Representative spectra for samples comprising hydrogels synthesized with the three different nozzle sizes and hydrogels synthesized with medium nozzle + swelled in 1X PBS for 24 h at 37 °C. **D** Phase angle shifts of the corresponding spectra for all samples as outlined in **C** and which negatively correlated with hydrogel storage moduli. ***p < 0.001 by one way analysis of variance and Tukey’s post hoc analysis (JMP). All data expressed as mean ± SD (*n* = 3).

PEMC sensors were submerged in hydrogels, including PBS swelled hydrogels and non-swelled hydrogels synthesized with three different nozzle sizes during injection (Fig. 4a), to assess the hydrogel viscoelastic properties through the fluid-structure interaction effect [14] (Fig. 4b). Resonant frequencies ranged from 48 to 59 kHz. According to Fig. 4c, the different nozzle sizes yielded similar sensor phase angles ranging from − 87.4° to − 87.0°. In contrast, swelled hydrogels synthesized with the medium nozzle yielded phase angles that were higher from − 86.5° to − 86.3°, indicating a relatively softer gel. The results shown by Fig. 4d indicated that there was no significant difference in the corresponding phase angle shift due to the different nozzles (− 2.1° to − 2.2°), while swelled hydrogels led to significantly higher phase angle shifts (− 1.3°). Since the sensor phase angle at resonance negatively correlated with the storage modulus of the surrounding material [5, 14], the nozzle size used for extrusion did not impact hydrogel stiffness under the parameter range investigated, while hydrogel swelling for 24 h caused hydrogel softening (reduction in shear storage moduli). Hydrogel synthesis with medium nozzles led to the lowest variation in phase angle shifts (Fig. 4d), and it was deemed the most optimal nozzle for generating uniform hydrogels.

### Ultrasound Imaging of Porcine Brain Tissue Surrogates

**Fig. 5:**
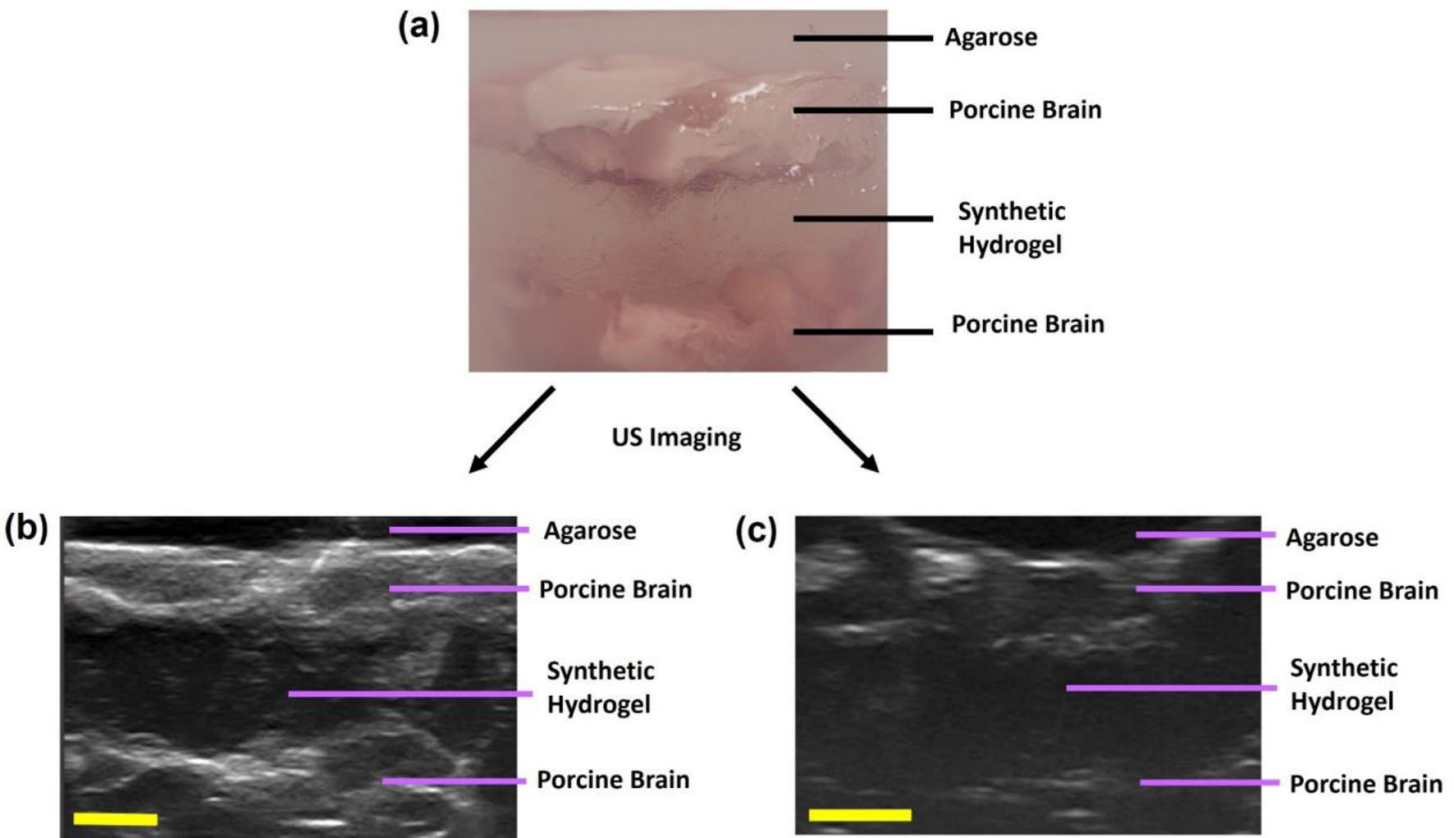
Ultrasound imaging of synthetic hydrogel in healthy porcine brain tissue surrogates. **A** Distinct layers in brain tissue surrogates within agarose resection phantoms. B-scan ultrasound images of the surrogates were obtained, with porcine brain tissues being hyperechoic (gray) and the synthetic hydrogels being hypoechoic (dark) for surrogates with cranial implant (**B**) and without cranial implant (**C**). Three replicates of porcine brain tissue surrogates were prepared for each condition. Scale bar is 5 mm.

Ultrasound imaging revealed the porcine brain tissues could be clearly visualized and distinguished from the synthetic hydrogel which was visualized as a central hypoechoic region (Fig. 5a**)**. Ultrasound imaging through the cranial implant led to some attenuation (Fig. 5b) compared to imaging without the cranial implant, with the overall image showing a lower signal (i.e. darker image), but the synthetic hydrogel was still distinguishable as a dark hypoechoic region.

### Histotripsy Cavitation Threshold

**Fig. 6:**
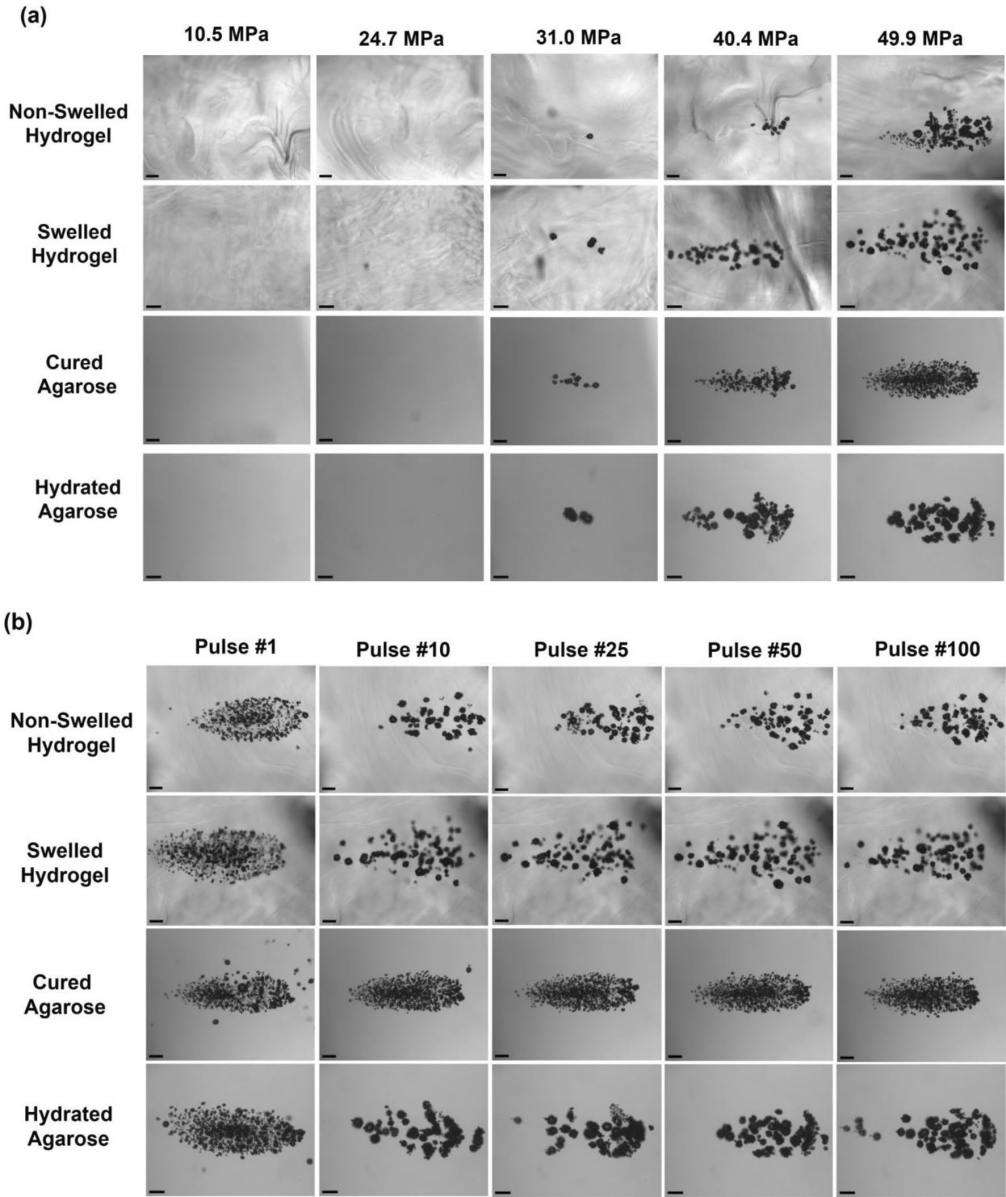
Cavitation bubble clouds during histotripsy treatment of swelled and non-swelled synthetic hydrogels and corresponding agarose hydrogel controls. Hydrogels were synthesized in the agarose resection phantoms and subjected to histotripsy treatment immediately (non-swelled) or submerged in 1X PBS at 37 °C for 24 h prior to treatment (swelled). For all treatments, 100 histotripsy pulses were applied at 0.5 Hz PRF with a 500 kHz transducer. Cured agarose hydrogels (as formed) and agarose hydrogels submerged in 1X PBS at 37 °C for 24 h (hydrated) were used as the corresponding control group for cavitation threshold studies. Focused ultrasound waves are being transmitted from right to left. **A** Representative cavitation bubble cloud images in hydrogels taken from the 50^th^ pulse at various peak negative pressures. **B** Representative cavitation bubble cloud images in hydrogels from a range of pulses at 49.9 MPa. Scale bars are 1 mm.

Representative cavitation bubble cloud images at select *p*− are shown by Fig. 6a for swelled/non-swelled synthetic hydrogels and the corresponding cured/hydrated control agarose hydrogels. Representative cavitation bubble cloud images at 49.9 MPa for a range of pulses are shown by Fig. 6b for swelled/non-swelled synthetic hydrogels and the corresponding cured/hydrated control agarose hydrogels. The clouds matched the focal region and formed dense bubble clouds with no cavitation bubbles observed outside of the central bubble cloud. Clouds were less dense after the first pulse, but remained well confined to the focal region, demonstrating histotripsy can be applied with high precision inside these synthetic hydrogels.

**Fig. 7:**
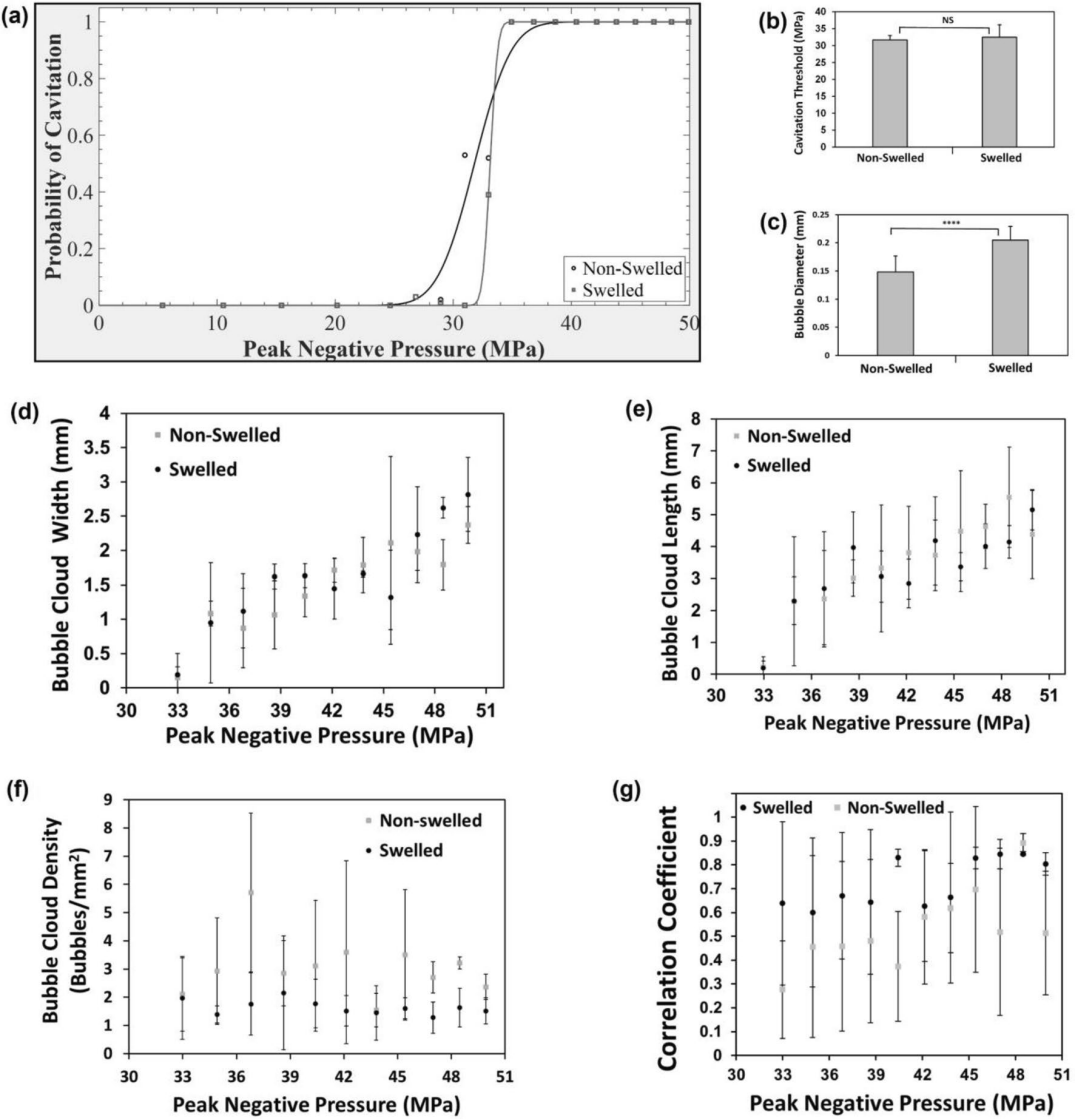
Quantification of bubble characteristics during histotripsy treatment of swelled and non-swelled synthetic hydrogels**. A** Representative cavitation threshold curves for swelled and non-swelled hydrogels based on probability of cavitation occurring against the peak negative pressure level. **B** Cavitation threshold of swelled vs non-swelled hydrogels based on 50% probability of cavitation occurring. **C** Average diameters of individual bubbles in swelled and non-swelled synthetic hydrogels for peak negative pressures above the cavitation threshold. Bubble cloud sizes in terms of **D** transverse width and **E** axial length measured for pulses at each peak negative pressure above the cavitation threshold for non-swelled and swelled hydrogels. **F** Bubble cloud density of swelled and non-swelled synthetic hydrogels for peak negative pressures above the cavitation threshold. **G** Correlation coefficient to quantify the pulse-to-pulse presence of persistent bubbles during cavitation. All data expressed as mean ± SD (*n* = 3).

Representative cavitation threshold curves (Fig. 7a) indicated similar sigmoidal curves for both swelled/non-swelled hydrogels, with swelled hydrogels possessing a much sharper rise in the cavitation threshold curve due to the presence of residual bubbles in the hydrogel increasing the probability of cavitation occurring near the *p−* where cavitation is likely to occur. The average cavitation threshold for the non-swelled hydrogels was 31.7 ± 1.3 MPa and not significantly different from that of the swelled hydrogels at 32.5 ± 3.6 MPa (Fig. 7b). Swelled hydrogels led to significantly higher individual bubble diameters at 0.205 ± 0.025 mm compared to non-swelled hydrogels at 0.148 ± 0.028 mm at timepoints when clouds were imaged (Fig. 7c). We had previously characterized the cavitation thresholds and bubble characteristics in 1% agarose hydrogels [10], which conform to our current study with agarose controls that yielded cavitation threshold pressures ranging from 29.0 to 29.5 MPa. The hydrated agarose yielded larger bubbles at 0.157 ± 0.048 mm compared to cured agarose hydrogels at 0.096 ± 0.022 mm. The bubble cloud size profiles for non-swelled/swelled hydrogels in terms of the cloud width (Fig. 7d) and cloud length (Fig. 7e) were also similar for *p*− above the cavitation threshold. The axial length of the clouds ranged from 0.2 mm to approximately 5 mm upon full cloud expansion at high *p*− of 48.5 MPa and above. Amongst the different hydrogels, the bubble clouds looked similar during the first histotripsy pulse (dense cloud of small bubbles) and for subsequent pulses within the same hydrogel, the bubble sizes increased and bubble density decreased. For *p−* above the cavitation thresholds, the bubble cloud density was higher for the non-swelled hydrogels compared to the swelled synthetic hydrogels (Fig. 7f). The pulse-to-pulse correlation coefficients (Fig. 7g) quantified persistent bubbles that expanded/collapsed in same location between pulses for swelled/non-swelled hydrogels at *p*− above the cavitation threshold. Swelled hydrogels had a higher coefficient of 0.73 ± 0.10 compared to 0.53 ± 0.16 in non-swelled hydrogels, likely due to the presence of more residual bubbles between pulses.

### Red Blood Cell Ablation

**Fig. 8:**
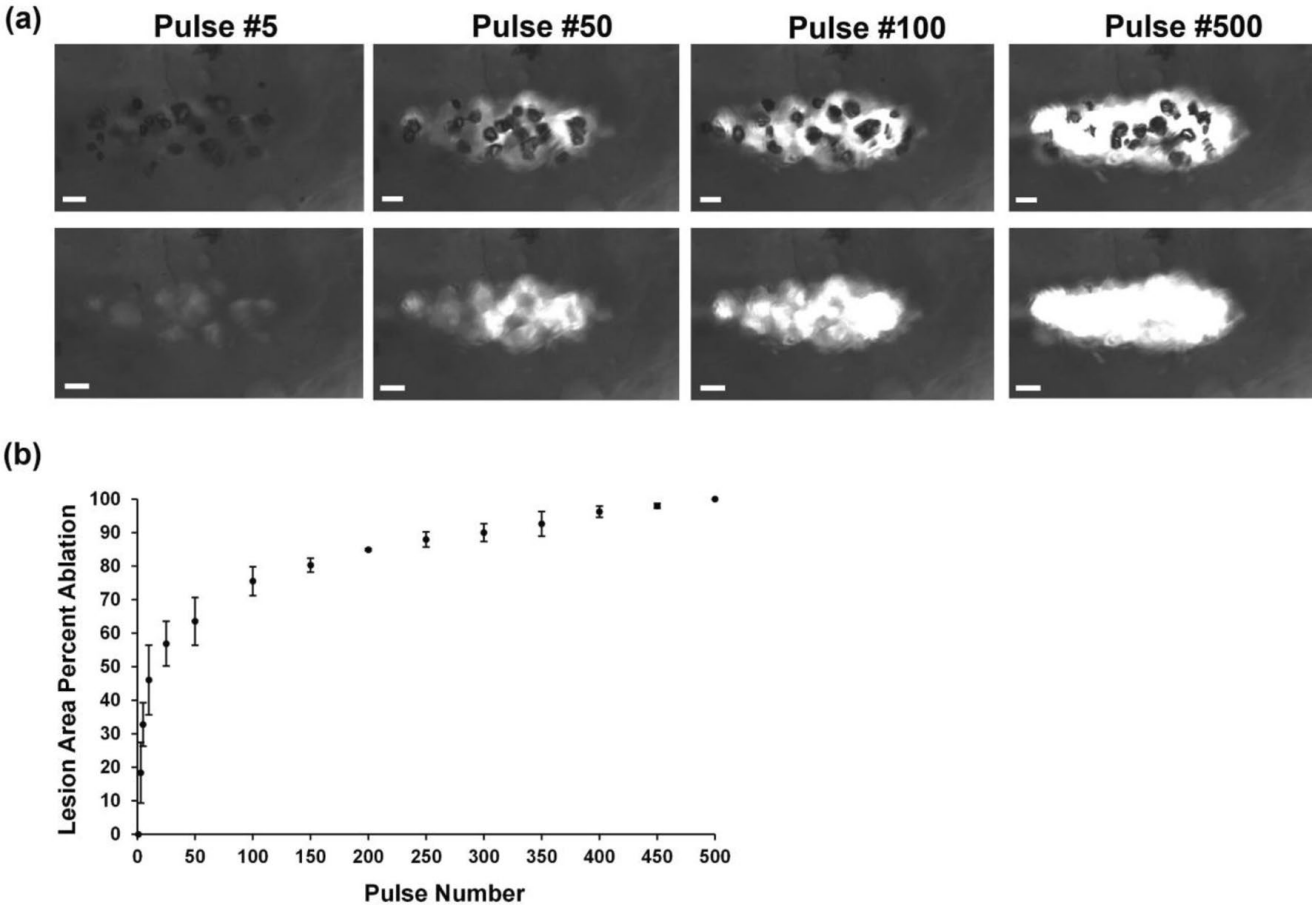
Red blood cell ablation with histotripsy treatment. **A** Representative images of the RBC at each pulse when cavitation bubble cloud formed (top layer) and immediately before the next pulse without the cavitation bubble cloud (bottom layer). Representative images shown for pulse 5, 50, 100, and 500 applied with 500 kHz transducer at 0.5 Hz PRF and at 48 MPa peak negative pressure. Scale bars represent 1 mm. Focused ultrasound waves are being transmitted from right to left. **B** Lesion area in terms of percentage ablated per pulse. All data expressed as mean ± SD (*n* = 3). At least three different regions were treated per hydrogel.

Histotripsy treatment of the RBC layer entrapped by the synthetic hydrogel layers demonstrated the lesion area was sharply demarcated and matched in shape and size to the cavitation bubble cloud, indicating the ablation zone was confined to the focal point where the cavitation occurred during pulsing (Fig. 8a). As indicated by Fig. 8b, nearly 65% of total ablation was achieved within the first 50 pulses. From 100 pulses and onwards, the ablation increased incrementally until near complete ablation was achieved at 500 pulses, with only a very small island of cells remaining within the focal volume after 500 pulses, resulting in nearly 100% of the focal region being effectively ablated.

## Discussion

**Fig. 9:**
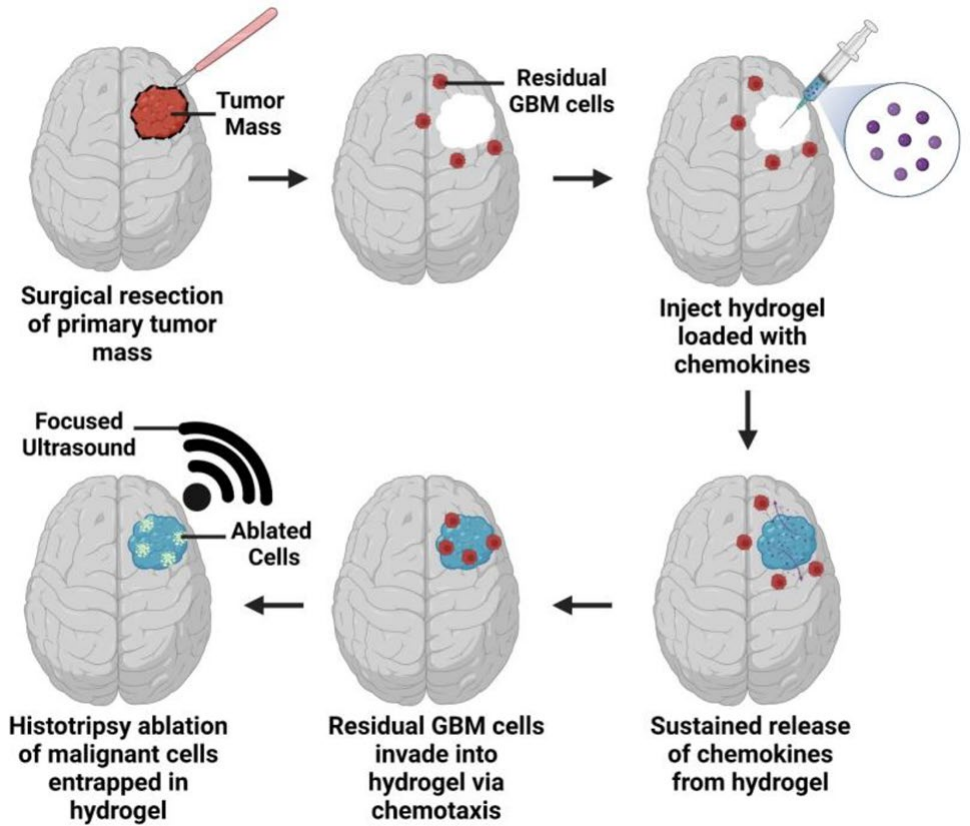
Schematic overview of proposed hydrogel-based glioblastoma cell entrapment strategy. Post-surgery after removal of the primary GBM tumor mass, a hydrogel preloaded with chemokines can be injected into the empty resection cavity for crosslinking *in situ*. Release of the chemokines from the hydrogel can then induce the migration of residual GBM cells via chemotaxis. After the malignant cells are localized and entrapped in the hydrogel, histotripsy can be applied to non-invasively and mechanically ablate the cells with focused ultrasound treatment. Images created with BioRender.com and adapted from our previous work [17, 18].

We previously determined synthetic hydrogels with 0.175 M NaHCO_3(aq)_ and 50 wt% hydration were the most optimal for physical, chemical, and biological compatibility with the GBM microenvironment [19], and sustained CXCL12 release from the hydrogels induced GBM invasion via chemotaxis [17]. In our current work, we synthesize this hydrogel in an agarose resection cavity and assess the feasibility of ablating hydrogel-entrapped cells with histotripsy. For future clinical application, we envision this synthetic hydrogel, upon injection into the GBM resection cavity and crosslinking *in situ* according to patient-specific anatomy, can capture and localize residual GBM cells into the hydrogel through chemotaxis for their subsequent mechanical ablation with histotripsy (Fig. 9).

In this study, we show the hydrogel crosslinks in 41 seconds [19] within a clinically relevant timeframe without clogging the nozzle. The hydrogels displayed promising bio-orthogonal properties upon direct injection into PBS, which mimicked crosslinking *in situ* at physiologic osmolarity, pH, and volume. Compared to rheometers that require time-consuming manual sample preparations and relatively large sample volumes, PEMC sensors obtain critical material properties using low volume, high-throughput measuring formats [14]. These sensors exhibit resonance in PEGDA-based hydrogels even after sol–gel transition, with a phase angle that negatively correlates with storage moduli [5, 14]. Thus, the resonant milli-cantilever phase angle response is equivalent to the output of a milli-scale rheometer. The behavior of sensor phase angle confirmed the synthetic hydrogel was not shear-thinning, as nozzle size did not impact the viscoelastic properties when hydrogels crosslinked via covalent bonding. Medium nozzles led to the least batch-to-batch variability (Fig. 4d), since they aligned with the diameters of commercial spiral static mixers and were therefore deemed the most optimal size. The PEMC sensors were able to detect the subtle difference between the swelled and non-swelled synthetic hydrogel, which indicated the sensor limit of detection [14] was within the range needed to characterize these hydrogels.

For ultrasound imaging of the hydrogels, porcine brain tissues were utilized as surrogates due to their similar white and gray matter to human brains [36]. The synthetic hydrogel was distinguishable from surrounding brain tissue by ultrasound imaging, which can enable patient-specific adjustments to histotripsy parameters for real-time monitoring of bubble clouds during treatment. Ultrasound imaging through the cranial implant distinguished the synthetic hydrogel from brain tissue. Prada and colleagues determined sonographic imaging through this implant mitigated ultrasound attenuation compared to imaging directly through skulls [29], as its low-porosity polymers lead to a uniform artificial structure and predictable interactions with ultrasound waves [29]. The cranial implant led to attenuation at maximum pressure and for 500 kHz transducers at the maximum input voltage, the pressure attenuation was reported to be − 0.484 dB, while the mean attenuation across a range of input voltages was − 0.567 ± 0.023 dB. While lower frequencies can be used during ultrasound imaging for improved penetration into deeper structures in the brain, higher frequencies such as those used in our study can be combined with the cranial implant to help improve spatial resolution during imaging. This cranial implant can potentially replace a portion of the skull for GBM patients requiring chronic monitoring to enable more frequent, low-cost imaging of GBM recurrence after surgery and potential delivery of FUS therapies with portable, ultrasound guided devices. Ultrasound imaging may visualize GBM cell infiltration into the hydrogels, which may guide histotripsy treatment and should be investigated in future research.

Since intrinsic threshold nucleation occurs within the water inside tissues [39], we assessed the impact of hydrogel swelling and determined there was no significant difference in cavitation threshold pressures between swelled and non-swelled hydrogels. Intrinsic histotripsy cavitation is reliant on nuclei intrinsic to the medium [23], and the hydrogel fluid properties did not change with swelling. Previous research showed tissue stiffness did not impact the intrinsic threshold for materials with Young’s moduli less than 1 MPa, because the cavitation threshold is dependent on the surface tension instead of the medium’s macroscopic viscoelastic properties [39]. Since the synthetic hydrogels possess stiffnesses in the kPa range as determined previously [19], the cavitation thresholds were not impacted by hydrogel swelling.

Swelled and non-swelled hydrogel cavitation thresholds conformed to the literature for soft, water-based tissues, as agarose hydrogels treated with transducer frequencies from 345 kHz to 3 MHz have thresholds between 25 and 30 MPa, similar to intrinsic cavitation thresholds for brain tissues [23, 39]. Agarose hydrogels do not swell [15], and although agarose hydration did not impact the cavitation thresholds, the data matched well with our previous studies in which we had extensively characterized the cavitation, bubble characteristics, and ablation capacities of agarose-based tissue phantoms [9, 10, 34]. Swelled hydrogels with a lower stiffness led to larger bubbles, since bubble sizes during histotripsy correlate with the magnitude of strain and stress bubbles exert upon expansion/collapse during cavitation [10], with stiffer tissues resulting in smaller bubble sizes [39]. Since the synthetic hydrogel was responsive to histotripsy, future research will investigate utilizing acoustic cavitation to release hydrogel therapeutic payloads toward controlling drug delivery [37] in GBM therapy. Future studies will explore the inclusion of acoustically active nanoparticles [10] for selective, precise hydrogel-encapsulated GBM ablation at lower acoustic pressures without needing ultrasound image guidance.

The results from Fig. 6b indicated over the course of the first few pulses, the bubble sizes increased in both the synthetic and agarose hydrogels. These results are consistent with prior histotripsy studies in other tissue-mimicking hydrogels due to the physical breakdown of the hydrogel as the bubbles ablated the hydrogel during the histotripsy treatment process. Although the bubble cloud sizes were the same, the bubble density in the non-swelled hydrogels was higher due to smaller sized bubbles, which led to a higher number of overall bubbles within the same given area. It should also be noted that the bubble numbers and cloud density analysis are likely an underestimate for clouds with a higher bubble density, as it is difficult to resolve and identify every individual bubble as determined in our previous studies [9, 38].

Ablation studies were performed specifically at *p*− of 48 MPa to ensure a full, expanded bubble cloud formed for complete ablation. Since histotripsy lesion areas in RBC phantoms align very well with lesion areas of *ex vivo* tissue phantoms according to histology [24], RBC phantoms were used for the ablation studies in this work. As illustrated by Fig. 8a, the cavitation bubble cloud was maintained throughout treatment for well-defined ablation zones aligning well with the clouds. The plot of lesion area percent ablation vs pulse number (Fig. 8b) demonstrated approximately 90% ablation was achieved within 300 pulses, which aligned with a previous study where 300 histotripsy pulses at low PRFs led to a complete cavity and breakdown in soft agarose hydrogels [26]. These findings further confirmed the hydrogel viscoelastic [19] and acoustic properties were tissue-mimicking.

The correlation coefficients of bubbles (Fig. 7g) indicated the presence of persistent residual cavitation nuclei between pulses in the synthetic hydrogels, which seed cavitation for subsequent pulses. These persistent cavitation nuclei were more prevalent in swelled hydrogels. This cavitation memory effect can prevent new bubbles from forming [10], as the incident acoustic field from each pulse may interact with existing nuclei to prevent cavitation at other sites within the focal volume [8], resulting in less efficient ablation within the treated area. While these persistent cavitation nuclei likely occurred due to higher gas concentrations introduced during synthesis and swelling in PBS, they did not impact ablation, as RBC ablation occurred uniformly in the hydrogels within the lesion area and ablation zone.

One limitation of our current study is we utilize RBCs for the ablation studies instead of GBM cells. Suspended RBCs undergo mechanical fractionation at smaller deformations than ECM-adhered cells, as changes to tissue stiffness with treatment affect the matrix, adhesion, and cells that in turn impact the tissue fracture strength and strain exerted by bubbles [40]. Hence, ablation of GBM cells encapsulated in the synthetic hydrogels could require slightly longer FUS treatment times for complete cell ablation. Ablation of GBM cells within the hydrogel will also lend important insights into whether histotripsy can launch a cell-mediated immune response within the otherwise immunosuppressive GBM tumor microenvironment, since histotripsy ablation of tumors can lead to a systemic, anti-tumor immune response through the release of proinflammatory cytokines and damage associated molecular patterns [16].

While our ablation zones aligned well with lesion areas of a previous *in vivo* porcine brain tissue histotripsy study [36], future research will investigate histotripsy of GBM cells encapsulated in the synthetic hydrogel and surrounded by ECM to recapitulate cell ablation at the hydrogel-ECM interface. Although soft brain tissue would dominate the viscoelastic properties of the resection cavity in the absence of GBM tissue *in vivo*, our *in vitro* histotripsy setup warranted agarose hydrogel phantoms that were stiffer than soft brain tissue, as stiff agarose-based resection phantoms ensured the synthetic hydrogels were securely in place during histotripsy treatment. Future research with synthetic hydrogels in an *in vivo* orthotopic rodent GBM resection model will investigate the impact of soft brain tissue at the interface of the hydrogel-tissue boundary during histotripsy treatment in the resection cavity. Another limitation is that the impact of histotripsy on hydrogel stability was outside the scope of the current study. Material toughness would indicate resistance to histotripsy ablation by considering the energy required until complete mechanical failure or fractionation [26]. Hence, future research should investigate ablation-induced structural damage to the material, which may be visualized with phase contrast microscopy [26] or quantified with rheology to assess stiffness reduction [42] for insights into the optimal histotripsy treatment duration.

In addition to the viscoelastic properties of the target medium, another limitation is that histotripsy treatment and ablation efficacies also depend on a range of other factors. For example, the transducer geometry and frequency impact the focal zone size, while the FUS parameters like PRF and *p−* impact the cavitation bubble dynamics, which all together can affect the number of histotripsy pulses that would need to be applied for complete ablation [45]. As such, future work is planned to develop ultrasound image-guided histotripsy systems for ablating GBM cells within the hydrogels through the cranial implants with patient-specific treatment parameters and feedback methods.

Overall, we demonstrated the thiol-Michael addition hydrogel synthesis can be scaled up for crosslinking with a dual chamber syringe mixer under conditions mimicking a resection cavity. The hydrogel was bio-orthogonal, not shear-thinning, and distinguishable by ultrasound imaging. Histotripsy treatment confirmed cavitation bubble clouds in the hydrogels can ablate encapsulated RBCs for uniform, well-defined lesions. The results demonstrate this synthetic hydrogel is a promising platform for histotripsy ablation toward GBM therapy.

## Abbreviations

3D: Three-dimensional
ECM: Extracellular matrix
FGPA: Field-programmable gate array
FUS: Focused ultrasound
GBM: Glioblastoma
p −: Peak negative pressure
PBS: Phosphate buffered saline solution
PEGDA: Poly(ethylene glycol) diacrylate
PEMC: Piezoelectric-excited millimeter cantilever
PLA: Polylactic acid
PRF: Pulse repetition frequency
RBC: Red blood cell
